# Primary osteosarcoma of the heart with long-term survival: A case report of laparoscopic resection of a metastatic sarcoma in the intestine

**DOI:** 10.3892/ol.2014.2405

**Published:** 2014-08-01

**Authors:** SHIKI FUJINO, NORIKATSU MIYOSHI, MASAYUKI OHUE, SHINGO NOURA, SHUICHI HAMAMOTO, KAZUYA OSHIMA, NOBUHITO ARAKI, YASUHIKO TOMITA, MASAHIKO YANO

**Affiliations:** 1 Department of Surgery, Osaka Medical Center for Cancer and Cardiovascular Diseases, Higashinari-ku, Osaka 537-8511, Japan; 2 Department of Orthopedic Surgery, Osaka Medical Center for Cancer and Cardiovascular Diseases, Higashinari-ku, Osaka 537-8511, Japan; 3 Department of Pathology, Osaka Medical Center for Cancer and Cardiovascular Diseases, Higashinari-ku, Osaka 537-8511, Japan

**Keywords:** osteosarcoma, heart, laparoscopic rectectomy, metastatic sarcoma

## Abstract

Primary cardiac tumors are uncommon and cardiac osteosarcoma is a rare disease. While complete surgical resection is considered to be the best treatment option for cardiac osteosarcomas, local and metastatic recurrences present challenges and indicate a poor prognosis. A combination of surgical resection with radio- and/or chemotherapy is a more effective course of treatment for osteosarcoma. In the present case, the patient underwent a complete resection of a primary cardiac osteosarcoma, and received chemotherapy and radiotherapy following local recurrence and metastasis to the bone post-operatively. Following these treatments, a rectal metastatic tumor was detected as causative of anemia. There is currently a lack of guidelines on the treatment of metastatic osteosarcomas in the intestine and there are few reports on rectal metastases. The present study described a laparoscopic resection of the osteosarcoma. The patient recovered without any complications and radiotherapy and chemotherapy were administered post-surgery to treat the bone metastases. The patient remained healthy at a follow-up examination, 61 months post surgery.

## Introduction

Primary cardiac tumors are rare, as demonstrated by their low frequency (0.001–0.03%) in an autopsy series (1,2), which indicated that 15–25% of the primary cardiac tumors were malignant (3,4). Sarcomas comprised two-thirds of these malignancies. The sarcoma subtypes, known as angiosarcomas and myxofibrosarcomas, are the most common cardiac sarcomas. Notably, a cardiac osteosarcoma is extremely rare (3–8). Surgical resection is the preferred treatment option for this type of sarcoma; however, even with surgical treatment, patients generally have a poor prognosis, with the median overall survival ranging from 6 to 12 months (5,8–10). An important and critical factor related to the severity of the prognosis, is the occurrence of distant metastasis. Bakaeen *et al* (11) reported that even if a partial surgical resection is performed, the combination of surgical resection and radio- and/or chemotherapy is effective (11). To the best of our knowledge, there have been no previous reports regarding the resection of a metastatic rectal tumor originating from an osteosarcoma of the heart and there are no published guidelines for the treatment of metastatic sarcomas in the intestine. The present case study discusses a patient who underwent a laparoscopic resection of a metastatic osteosarcoma in the rectum originating from the heart.

## Case report

**Figure 1:**
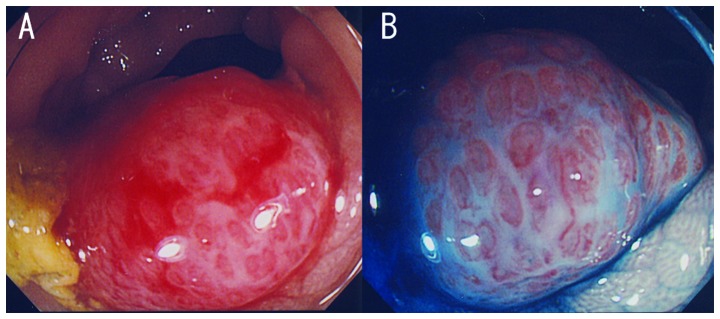
Preoperative colonoscopy. The surface color was red and the pit-pattern showed an irregular pattern, which differed from that of an adenocarcinoma.(A) Normal image and (B) indigo carmine-stained image.

**Figure 2:**
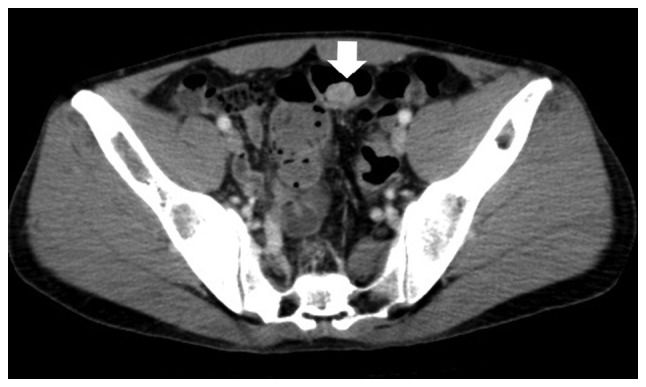
Preoperative contrast-enhanced computed tomographic scan of the abdomen. The arrow indicates an enhanced mass (15×14 mm) in the rectum.

A 41-year-old male was admitted to the Osaka Medical Center for Cancer and Cardiovascular Diseases (Osaka, Japan) with paraplegia and anemia in March 2013. The medical history of the patient indicated that a surgical resection of a cardiac osteosarcoma had been performed at another hospital in September 2008. In May 2010, the patient presented with heart failure and recurrence of the sarcoma in both atria. Subsequently, the patient underwent a second surgical resection for the recurrent heart sarcoma, together with radiotherapy of the heart, and chemotherapy consisting of adriamycin (ADR), ifsofamide (IFO) and gemcitabine (GEM), post-operatively. Positron emission tomography-computed tomography (PET-CT) revealed multiple bone metastases in July 2011, and therefore radiotherapy to the mandible and scapula, Sr89, and Pazopanib were added to the treatment regimen. In December 2012, magnetic resonance imaging revealed metastases in the thoracic vertebrae, causing paraplegia to develop in March, 2013. Radiotherapy to the thoracic vertebrae Th4-9 and chemotherapy (ADR, IFO) were administered to treat the metastases, together with nerve decompression surgery to the thoracic spinal nerves of the Th4, Th9 vertebrae. Although the paralysis was controlled by these treatments, the patient was anemic, as indicated by a blood test. A fecal occult blood test (FOB-test) was performed to determine the cause of the anemia and the positive results predicted a rectal tumor. Colonoscopy revealed a rectosigmoid tumor as the cause of the bleeding, but this tumor did not have a pit-pattern typical of rectal cancer (Fig. 1). Contrast-enhanced computed tomography (CT) showed the tumor in the rectum with no metastatic lymph nodes (Fig. 2). Additionally, the tumor exhibited a high fluorodeoxyglucose uptake, similar to other bone metastases, in PET-CT imaging.

**Figure 3:**
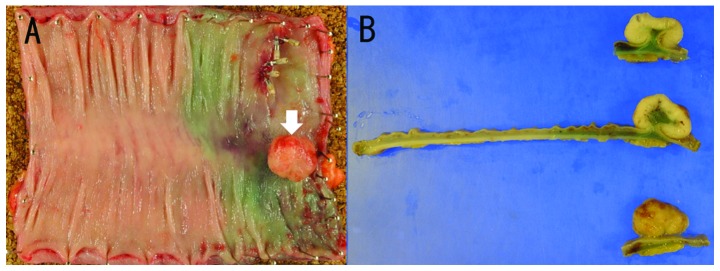
Macroscopic image of a metastatic osteosarcoma in the rectum. A 15×15 mm mass was located in the rectum. (A) Fresh tissue and (B) fixed by formaldehyde.

**Figure 4:**
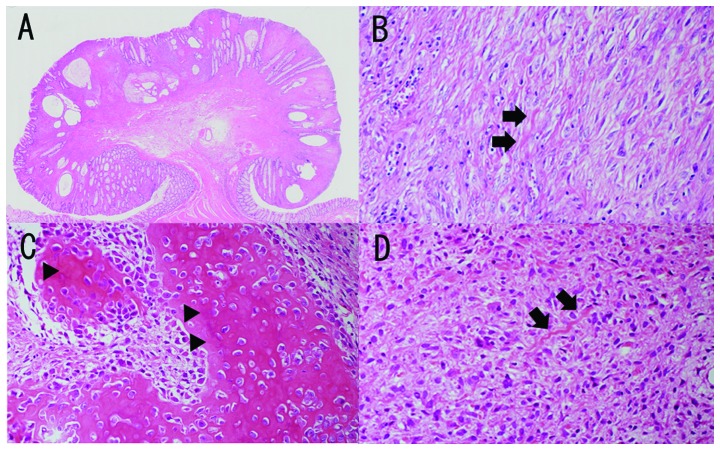
Photomicrograph of metastatic osteosarcoma in rectum and heart. (A and B) Photomicrograph of a metastatic osteosarcoma in rectum. Polygonal and spindle cells with an eosinophilic cytoplasm and an osteoid matrix were observed (shown by arrows). (C and D) Photomicrograph of a heart osteosarcoma. The bone formations (shown by arrow heads in C) and eosinophilic osteoid (shown by arrows in D) were observed.

Histological examination of the biopsy sample revealed growth of spindle cells with irregular nuclei. Immunohistochemical examination showed positive staining for Ki67 in 5–10% of the cells. These findings were compatible with a histological diagnosis of a metastatic osteosarcoma. To avoid bleeding from the tumor and future obstruction of the intestine, a laparoscopic rectectomy following indocyanine green marking (12) was performed to completely remove the rectal tumor (Fig. 3). Histological examination of this tumor specimen revealed dense groups of polygonal and spindle cells, with eosinophilic cytoplasms and pleomorphic nuclei with high density chromatin and irregular nucleoli. The osteoid matrix did not appear to have any calcification and there was no lymph node metastasis. These pathological findings were also detected in the cardiac osteosarcoma sample (Fig. 4). Thus, these findings were compatible with a histological diagnosis of a metastatic rectal osteosarcoma originating from the heart.

The patient recovered without any complications, and radiotherapy to thoracic vertebrae 12 to lumba vertebrae 5, and chemotherapy with ADR and cisplatin were administered post-operatively to treat the bone metastases. The patient was discharged from the hospital on postoperative day 67 and remained alive 61 months after the initial operation. The patient is still receiving chemotherapy and radiotherapy for the bone metastases.

## Discussion

**Figure 5:**
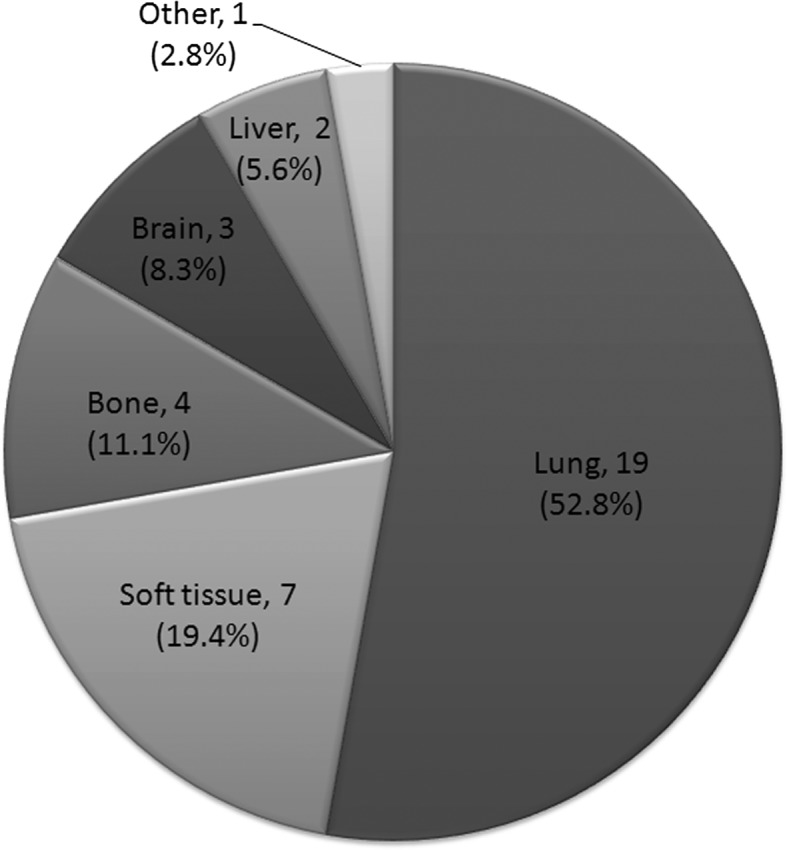
Regions of metastases derived from a cardiac sarcoma. Regions of metastases of the cardiac sarcoma were reported in lung (19 cases), soft tissue (7 cases), bone (4 cases), brain (3 cases), liver (2 cases) and abdomen (1 case, unknown).

Life-threatening consequences of primary malignant cardiac tumors include obstruction to the intracardiac blood flow, interference to valve function, arrhythmias, and pericardial tamponade resulting from local invasion (5,8,13–17). Complete surgical resection, whenever possible, is considered the best treatment option (5–11) since it has been associated with an improved survival period (17 months after complete surgical resection, as compared with 6 months without resection) (5). In the majority of cases, the patients develop a local recurrence and metastases following the initial surgical resection. Under these conditions, several reports have shown that effective palliation of local recurrences are possible and effective (8–11,18,19). Metastasis at the time of presentation has an impact on the prognosis; the median survival period of patients with metastases has been reported to be 5 months, as compared with 15 months, in patients without metastases (5). Additional therapies, such as chemotherapy and/or radiotherapy, improve the prognosis of patients with metastatic diseases and those with a local recurrence (9). Reported regions of metastases of the cardiac sarcoma (Fig. 5) (5,7,19) include the lungs, which are the most common site, in addition to soft tissue (including the mediastinum), bone, brain, and liver. The lung, thyroid, brain, intestine, peritoneum, bone, and skin were previously reported as metastatic regions of cardiac osteosarcoma, with a median survival time of 9 months (range, 0–67 months) (13–17). The present study, to the best of our knowledge, reports the first case of a metastasis to the rectum, which was surgically resected and the patient experienced a prolonged survival as previously defined.

The patient in this case has been alive for 61 months following the first surgical resection, and 37 months after the presentation of multiple metastases. This is considered long-term survival. Upon careful analysis, this study proposes that repeating the surgical resection is the optimum choice of treatment as it is necessary to remove the rectal metastasis to prevent anemia and future obstruction To the best of our knowledge, this is the first reported case of using laparoscopic resection of a rectal metastasis. Laparoscopic surgery is minimally invasive and feasible for this type of metastatic tumor. In the present case, the patient recovered without any complications and was able to undergo chemotherapy and radiotherapy shortly after the repeated surgical resection. The literature on sarcoma metastases to the intestine is limited and there are no reports on metastases to the rectum (8). Data from the present case study suggest that where a FOB-test is positive in the sarcoma patient, it is necessary to perform a colonoscopy in order that a metastatic lesion is not overlooked. Adjuvant chemotherapy and radiotherapy remain controversial treatment options for patients who have undergone a complete surgical resection, and there is no established approach for the treatment of patients with metastases (13–19). In this case, complete resection for the primary cardiac osteosarcoma was performed, although recurrent local disease and metastases developed later. The patient underwent chemotherapy and radiotherapy for the bone metastases and surgical resection of the rectal metastasis. The patient remains alive after ~5 years since the first operation.

In conclusion, it is suggested that aggressive, complete surgical resection with radio- and/or chemotherapy is an effective course of treatment for osteosarcomas. This combination therapy can provide a palliative choice and leads to an improved prognosis for patients, even those with metastatic sarcomas.
